# Symphony of Well-Being: Harmony Between Neural Variability and Self-Construal

**DOI:** 10.3389/fnhum.2021.679086

**Published:** 2021-06-30

**Authors:** Leyi Fan, Qin Duan, Siyang Luo

**Affiliations:** ^1^Department of Psychology, Guangdong Key Laboratory of Social Cognitive Neuroscience and Mental Health, Sun Yat-sen University, Guangzhou, China; ^2^Department of Psychology, Guangdong Provincial Key Laboratory of Brain Function and Disease, Sun Yat-sen University, Guangzhou, China

**Keywords:** life satisfaction, neural variability, interdependence, default mode network, self-construal

## Abstract

Both neural activities and psychological processes vary over time. Individuals with interdependent self-construal tend to define themselves and adjust their behaviors to social contexts and others. The current research tested the hypothesis that the coordination between interdependent self-construal and neural variability could predict life satisfaction changes in university freshmen. We integrated resting-state functional magnetic resonance imaging scanning and self-construal assessment to estimate self-dependent neural variability (SDNV). In the whole-brain prediction, SDNV successfully predicted individuals’ life satisfaction changes over 2 years. Interdependent individuals with higher neural variability and independent individuals with lower neural variability became more satisfied with their lives. In the network-based prediction, the predictive effects were significant in the default mode, frontoparietal control, visual and salience networks. The important nodes that contributed to the predictive models were more related to psychological constructs associated with the social and self-oriented functions. The current research sheds light on the neural and psychological mechanisms of the subjective well-being of individuals from a dynamic perspective.

## Introduction

Life satisfaction, the cognitive component of subjective well-being (Andrews and Withey, 1976; Diener et al., 1985) and an important dimension of mental health (Headey et al., 1993), is based on a general evaluation of the life situations of individuals according to their own standards (Shin and Johnson, 1978; Diener et al., 1985). Life satisfaction has been strongly associated with positive and advantageous outcomes, including more social support, more positive social relationships, and better academic and work performance (Diener and Seligman, 2002; Guney, 2009; Erdogan et al., 2012). The neural substrates of life satisfaction have been studied from a static perspective (Kong et al., 2015a,c; Kim et al., 2016). In a resting-state functional magnetic resonance imaging (rs-fMRI) study, life satisfaction was positively correlated with the regional fractional amplitude of low frequency fluctuations (fALFF) in the left postcentral gyrus, bilateral posterior superior temporal gyrus, and left planum temporale but negatively correlated with the fALFF in the bilateral superior frontal gyrus (Kong et al., 2015c). In a structural magnetic resonance imaging study, the regional gray matter volume (rGMV) in the right parahippocampal gyrus positively predicted life satisfaction, whereas the rGMV in the left ventromedial prefrontal cortex and left precuneus negatively predicted life satisfaction (Kong et al., 2015a). However, life satisfaction changes over time and is based on different trends among individuals (Mroczek and Spiro, 2005; Hope et al., 2014). Thus, it is necessary to investigate the neural mechanism of life satisfaction from a dynamic perspective.

Apart from psychological processes, neural activities also vary over time. Neural dynamics are shown on different temporal and spatial scales. Over time, neuroplasticity, which involves changes in the structures and functions of the brain to adapt to the environment, describes neural dynamics in the long term. Neuroplasticity is associated with sensory and cognitive functions, including learning and memory (Münte et al., 2002; Dayan and Cohen, 2011), as well as mental disorders (Kalivas and O’Brien, 2008; Lewis and González-Burgos, 2008; Pittenger and Duman, 2008). Recently, there has been increasing interest in spontaneous neural fluctuations over short periods of time, especially dynamic functional connectivity (Hutchison et al., 2013; Zalesky et al., 2014; Liao et al., 2015; Preti et al., 2017). Functional connectivity characterizes the functional interdependence of brain regions (Biswal et al., 1995). In space, most studies on dynamic functional connectivity have been based on connectivity patterns across the whole brain at the macroscale (Allen et al., 2014; Liao et al., 2015; Choe et al., 2017) or between a pair of regions at the microscale (Chang and Glover, 2010; Zhang et al., 2018). Zhang et al. (2016) proposed a novel analysis at the mesoscale, gathering global and local information, which is called neural variability in the current research. Neural variability is the temporal variability of functional connectivity between a given region and all regions across the brain (Zhang et al., 2016).

Neural variability is altered in mental disorders, including schizophrenia (Guo et al., 2018; Deng et al., 2019; Dong et al., 2019; Long et al., 2020; Zhang et al., 2016), major depressive disorder (Hou et al., 2018; Sheng et al., 2018; Chen et al., 2019), bipolar disorder (Long et al., 2020), attention deficit hyperactivity disorder (Zhang et al., 2016; Zou and Yang, 2019) and autism spectrum disorders (Zhang et al., 2016). For instance, in one study, neural variability in the left inferior occipital gyrus was stronger in major depressive disorder patients than in healthy controls (Hou et al., 2018). Recent studies on neural variability have mainly focused on its predictive effects on the negative side of mental health (i.e., mental disorders). However, researchers have also suggested that neural variability might be an indicator of brain flexibility and adaptability (Zhang et al., 2016), which might in turn predict positive changes in mental health. We address this issue in the current research by examining its predictive effects on life satisfaction changes.

Moreover, social relationships and cultural circumstances play a role in shaping life satisfaction (Mroczek and Spiro, 2005; Cheng et al., 2011; Kong et al., 2015b; Zou et al., 2015). For example, social networks (Lim and Putnam, 2010) and social support (Heintzelman and Bacon, 2015) were found to be associated with life satisfaction. These findings imply that life satisfaction can be modulated by the response to the sociocultural environment of individuals (Lim and Putnam, 2010). A possible indicator of the social response is self-construal, which describes the self-concept from a sociocultural perspective. Self-construal is the self-definition and interpretation of individuals (Markus and Kitayama, 1991; Singelis, 1994). Interdependent self-construal, which is dominant in Eastern collectivistic cultures, defines the self-according to social contexts and others. Independent self-construal, which is dominant in Western individualistic cultures, defines the self as an autonomous and bounded entity (Markus and Kitayama, 1991). Self-construal modulates various cognitive/affective processes and their neural substrates (Han and Humphreys, 2016), including self-reflection (Ma et al., 2014), autobiographical memory (Wang, 2001), theory of mind (Ray et al., 2010), empathy (Jiang et al., 2014), and the occurrence and consequences of social comparison (Kemmelmeier and Oyserman, 2001; Stapel and Koomen, 2001; White et al., 2006), which enable individuals to compare their own present life situations with those of the past and of others, in turn affecting life satisfaction. Therefore, we hypothesize that self-construal moderates the relationship between neural variability and life satisfaction changes.

In addition, given that brain function has been implemented through systems and networks (Power et al., 2011; Bassett and Sporns, 2017), the question of which networks contribute to the prediction is raised. The default mode network is a set of brain regions whose activity increases during the resting state, including the medial prefrontal cortex and posterior cingulate cortex (Raichle et al., 2001). The default mode network is involved in self-related processes (Qin and Northoff, 2011; Wang et al., 2013), autobiographical memory (Spreng et al., 2009; Spreng and Grady, 2010), theory of mind (Spreng et al., 2009; Spreng and Grady, 2010; Li et al., 2014) and empathy (Li et al., 2014). Given the important role of the DMN in self-related processes and social processes, we expected that the DMN would contribute to the prediction of life satisfaction changes.

In the current research, we hypothesize that self-construal moderates the relationship between neural variability and life satisfaction changes. We perform rs-fMRI on university freshmen to estimate their neural variability. Self-construal is assessed using the Self-Construal Scale (Singelis, 1994), and life satisfaction is assessed two times using the Satisfaction with Life Scale (Diener et al., 1985) to obtain life satisfaction change scores. Finally, we use the leave-one-out cross-validation method to model the predictive effects. Specifically, university freshmen are chosen as the sample since they are experiencing the transition from adolescence to adulthood, adapting to a new environment, and developing new academic skills and social relationships (Boujut and Bruchon-Schweitzer, 2009), which might give rise to life satisfaction changes (Hope et al., 2014; Zhou and Lin, 2016).

## Materials and Methods

### Participants

Sixty-one healthy university freshmen (42 males, 19 females, age = 20.11 ± 2.33 years) participated in the study. Written informed consent was obtained from all participants before commencing the studies. All studies were approved by the ethics committee of the Department of Psychology at Sun Yat-sen University. To detect a robust predictive effect [95% confidence interval (CI) larger than zero] on individualized behavioral prediction with functional connectivity features, a sample of 60 participants was required (Cui and Gong, 2018).

### Behavioral Assessments

#### Self-Construal

**FIGURE 1:**
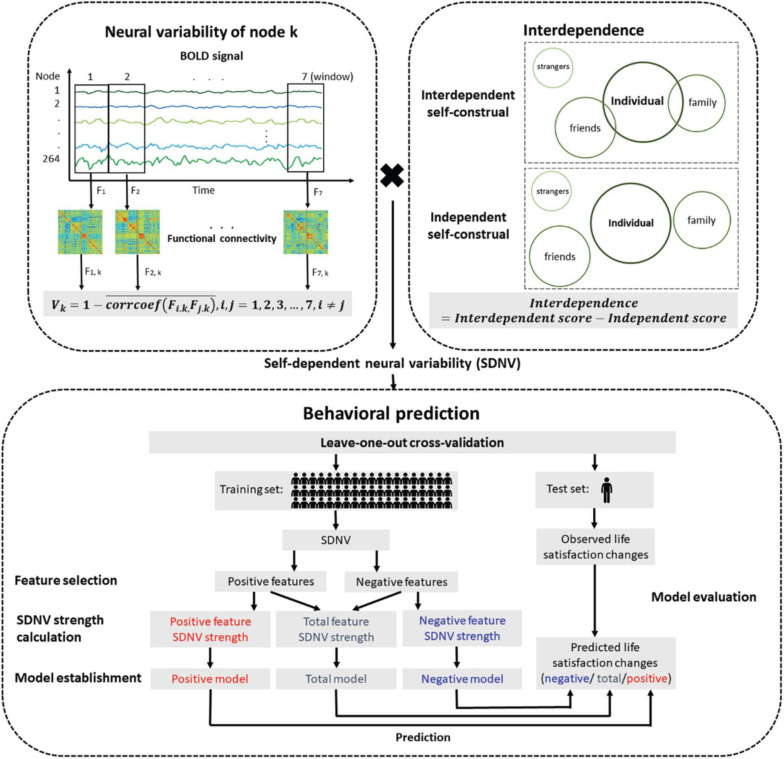
Flow chart of the prediction analysis. The neural variability of a node was the temporal variability of functional connectivity between the node and all nodes across the whole brain. Interdependent self-construal was the difference in the mean score between the interdependent self-construal subscale and the independent self-construal subscale. The self-dependent neural variability (SDNV) of a node was the interaction (i.e., dot product) between the neural variability of the node and interdependent self-construal. The leave-one-out cross-validation method was used to study whether SDNV could predict life satisfaction changes.

The participants completed the Self-Construal Scale (Singelis, 1994). This scale is divided into two dimensions: an interdependent subscale and an independent subscale. Each subscale includes 12 items that are rated on a 7-point Likert scale (1 = strongly disagree, 7 = strongly agree). Interdependent self-construal was assessed by subtracting the mean score on the independent subscale from the mean score on the interdependent subscale (Figure 1; Luo et al., 2015). Higher levels of interdependent self-construal indicated more interdependence in social contexts and others.

#### Life Satisfaction

The participants completed the Satisfaction with Life Scale (Diener et al., 1985). This scale includes 5 items that are rated on a 7-point Likert scale (1 = strongly disagree, 7 = strongly agree). The participants completed the Satisfaction with Life Scale twice over an approximately 2-year period: the first assessment was within the first month after they enrolled in the university, and the second assessment was approximately 2 years later (mean interval = 25.8 ± 0.24 months). Life satisfaction change scores were assessed by subtracting the life satisfaction scale score at the first assessment from the score at the second assessment. The life satisfaction change scores of three participants were missing; thus, they were excluded from the prediction analyses of life satisfaction change scores. Fifty-eight participants (41 males, 17 females, age = 20.10 ± 2.31 years) remained in the prediction analyses on life satisfaction changes.

The participants completed a demographic information survey that included their place of origin (urban-rural: 1 = large city, 2 = medium-sized city, 3 = small city, 4 = county, 5 = town, and 6 = village) and the Multigroup Ethnic Identity Measure (Phinney, 1992).

### Data Acquisition

We used a GE Signa MR750 3.0T scanner with a standard head coil to acquire rs-fMRI data. The resting-state data were acquired using T2-weighted, gradient-echo, echo-planar imaging (EPI) sequences with the following parameters: repetition time (TR) = 2,000 ms, echo time (TE) = 30 ms, flip angle = 90°, field of view (FOV) = 240 × 240 mm, matrix = 64 × 64 × 32, spatial resolution = 3.75 × 3.75 × 5 mm^3^, slice number = 32, and time duration = 300 s. The participants were instructed to keep their eyes open during scanning.

### Data Preprocessing

A standard preprocessing procedure was performed on the resting-state data using the Data Processing Assistant for Resting-State fMRI (DPARSF) toolbox (Yan and Zang, 2010). The data of the first five volumes were removed. The remaining data underwent slice timing and realignment to correct the time delay of scans and head motion. In the realignment procedure, no participant was excluded from the following analyses because all data were within the criterion of 3.0 mm and 3.0° maximum head motion. The corrected data were registered to Montreal Neurological Institute (MNI) space with an EPI template. The normalized data were smoothed with a 4-mm full width at half maximum (FWHM) Gaussian kernel, detrended, and bandpass filtered (0.01–0.08 Hz). Finally, nuisance covariates were removed by multiple regression, including six rigid-body head motion parameters and the mean time courses of white matter and cerebrospinal fluid.

### Parcellation Scheme and Network Definition

We used a 264-node atlas (Power et al., 2011) to define nodes and divided the nodes into 14 networks. The atlas included the cerebral cortex, subcortical structures and cerebellum. It contained 264 10-mm-diameter spheres that were defined as nodes. The center of the nodes was identified through two methods: meta-analyses based on task data and the mapping of cortical areas based on resting-state data. The nodes were divided into 14 networks: the auditory network, cerebellar network, cingulo-opercular task control network, default mode network, dorsal attention network, frontoparietal task control network, hand sensory-somatomotor network, memory retrieval network, mouth sensory-somatomotor network, salience network, subcortical network, uncertain network, ventral attention network, and visual network.

### Estimation of Neural Variability

We used the method proposed by Zhang et al. (2016) to estimate neural variability. The neural variability of a node was defined as the temporal variability of functional connectivity between the node and all nodes across the whole brain (Figure 1). Time series were extracted and split into seven nonoverlapping windows with a length of 40 s. A previous study suggested that the results with window lengths of approximately 30–60 s in duration were robust (Hutchison et al., 2013). Within window i, the 264 × 264 whole-brain functional connectivity matrix *F*_i_ was calculated using Pearson correlation analysis. The 264 × 1 vector *F*_i, k_ (i.e., row *k* or column *k* of *F*_i_) represents functional connectivity between node *k* and all nodes across the whole brain. For node *k*, the functional connectivity vectors in each pair of windows were compared using Pearson correlation analysis. The correlation coefficients of all pairs of windows were averaged. The neural variability of node *k* was calculated by subtracting the averaged correlation coefficient from 1. The formula is as follows:

$${\text{NV}}_{k}=1-\bar{\text{corrcoef}\,\left(F_{i.k},F_{j.k}\right)},i,j=1,2,3,\ldots ,7,i\neq j.$$

In addition, we estimated neural variability using different window lengths (window length = 30, 40, and 50 s) to test the robustness of the predictive effects across window lengths.

### Prediction Analysis

#### Whole-Brain Prediction

The self-dependent neural variability (SDNV) of each node was defined as the interaction (i.e., dot product) between the normalized neural variability of the node and normalized interdependent self-construal. We used the leave-one-out cross-validation method to study whether SDNV could predict the life satisfaction change score of a novel individual. The prediction analysis was divided into the following stages: feature selection, SDNV strength calculation, model establishment, prediction and model evaluation. The stages from feature selection to prediction formed an iteration. In each iteration, one participant was excluded to serve as the test set, and the remaining participants served as the training set. The training set established the predictive models, and the test set evaluated the predictive models. Because each of the 58 participants was excluded once, there were 58 iterations.

In the feature selection stage, we performed Pearson correlation analysis between the SDNV of each node and life satisfaction change scores in the training set (feature selection threshold ∝ = 0.05). The nodes whose SDNV was significantly positively correlated with life satisfaction change scores were allocated to the positive feature set, and the nodes whose SDNV was significantly negatively correlated were allocated to the negative feature set (feature selection threshold ∝ = 0.05). In addition, we used different feature selection thresholds (∝ = 0.10, 0.05, 0.01) to test the robustness of the predictive effects across the feature selection thresholds.

In the SDNV strength calculation stage, the SDNV values of the positive features or the opposite number of the SDNV values of the negative features were averaged, resulting in the positive feature SDNV strength or the negative feature SDNV strength, and the values of these two strengths were averaged together, resulting in the total feature SDNV strength. The formulas are as follows:

$$\mathrm{Total}\,\mathrm{feature}\,\mathrm{SDNV}\,\mathrm{strength}=\bar{{\text{SDNV}}_{k}\,m_{k}},$$

$$\mathrm{Positive}\,\mathrm{feature}\,\mathrm{SDNV}\,\mathrm{strength}=\bar{{\text{SDNV}}_{k}\,m_{k}^{+}},$$

$$\mathrm{Negative}\,\mathrm{feature}\,\mathrm{SDNV}\,\mathrm{strength}=\bar{{\text{SDNV}}_{k}\,m_{k}^{-}},$$

Where *k* indexed the node, *m*_k_ equaled 1 in the positive feature set and −1 in the negative feature set.

In the model establishment stage, we used simple linear regression to construct the relationships between each of the three SDNV strengths and life satisfaction change scores, resulting in three models: the total model, positive model, and negative model. The formulas are as follows:

$$\begin{array}{l} \text{Total model}:\text{Predicted life satisfaction change score}\\ \;=\text{Slope}\times \text{Total feature SDNV strength}+\text{Intercept}, \end{array}$$

$$\begin{array}{l} \text{Positive model}:\text{Predicted life satisfaction change score}\\ \;=\text{Slope}\times \text{Positive feature SDNV strength}+\text{Intercept}, \end{array}$$

$$\begin{array}{l} \text{Negative model}:\text{Predicted life satisfaction change score}\\ \;=\text{Slope}\times \text{Negative feature SDNV strength}+\text{Intercept}, \end{array}$$

In the prediction stage, based on the test set, the same features as the training set were extracted, and the three SDNV strengths were substituted in the three models, resulting in a predicted life satisfaction change score in each model.

In the model evaluation stage, the Pearson correlation coefficient between the life satisfaction change scores predicted by each model and the observed life satisfaction change scores measured by the scale was defined as the predictive power of the model. Only significantly positive predictive power indicated that the prediction was successful. When no feature was selected in at least one iteration, we did not perform the prediction analysis to maintain the consistency of the iteration number. The nodes that were selected as features in more than 95% of the iterations were regarded as important nodes.

The process is presented in Figure 1.

##### Partial Prediction

To control for the effects of neural variability and interdependent self-construal, we added the two variables as regressors in the feature selection stage and in the model establishment stage. In the feature selection stage, we used multiple regression analysis instead of Pearson correlation analysis in which SDNV, neural variability and interdependent self-construal were regressors. The nodes whose regression coefficient of SDNV was significant were selected as features. Neural variability strength was calculated with the same features and in the same way as the SDNV strength calculation. In the model establishment stage and in the prediction stage, we used multiple regression analysis instead of simple linear regression analysis in which SDNV strength, neural variability strength and interdependent self-construal were regressors. The predictive power of the three models indicated the survival of the predictive effects.

##### Permutation Tests

To further confirm the significance of the predictive effects, we performed permutation tests. In each permutation, we shuffled the life satisfaction change scores and reran the prediction analyses. Permutations were repeated 1,000 times in the three models. Thus, we generated a null distribution of the predictive effects for each model. Permutation tests provided evidence of how likely the predictive effects were observed at random.

### Network-Based Prediction

To study the contribution of specific networks, we used the nodes within each network instead of the nodes across the whole brain to perform the prediction analyses. In other words, only the neural variability of within-network functional connectivity was taken into account. Prediction analyses of the uncertain network were not performed because the function of the uncertain network was unspecified or unknown.

### Meta-Analytic Decoding

To understand the structural location and psychological function related to the important nodes in whole-brain prediction and in network-based prediction, we performed meta-analytic decoding using the Neurosynth Image Decoder (Yarkoni et al., 2011). We chose the important nodes in the default mode network to represent the network-based prediction. We pooled the important nodes or the remaining nodes together as a map and generated four maps: the important nodes in the whole-brain prediction, the remaining nodes in the whole-brain prediction, the important nodes in the default mode network-based prediction, and the remaining nodes in the default mode network-based prediction. The Neurosynth Image Decoder enabled us to compare each map to meta-analytic images related to various psychological constructs in the Neurosynth database.

### Specificity of the Predictive Effects

We tested the specificity of SDNV or life satisfaction change scores in the predictive effects.

#### Effects of Neural Variability or Interdependent Self-Construal

We tested whether neural variability or interdependent self-construal could predict life satisfaction change scores. To test the effects of neural variability, we used neural variability instead of SDNV to perform the prediction analyses. To test the effects of interdependent self-construal, we conducted Pearson correlation analysis between interdependent self-construal and life satisfaction change scores.

#### Effects on the Life Satisfaction Score at the First and Second Assessment

We tested whether SDNV could predict the life satisfaction score at the first assessment or the life satisfaction score at the second assessment. We used the score at the first assessment and the score at the second assessment instead of life satisfaction change scores to perform the prediction analyses.

#### Effects on Other Dependent Variables

We tested whether SDNV could predict variables that were independent of life satisfaction change scores, including the place of origin (urban-rural, independent objective information) and ethnic identity (independent subjective values) of individuals. We used the place of origin or ethnic identity instead of life satisfaction change scores to perform the prediction analyses.

#### Effects of Head Motion

We tested whether head motion patterns were correlated with life satisfaction change scores using Pearson correlation analysis to exclude the effects of head motion. We used six rigid-body parameters and framewise displacement to estimate head motion patterns with the DPARSF toolbox (Yan and Zang, 2010). The six rigid-body parameters reflected head motion patterns with reference to the first volume, including three translation parameters and three rotation parameters. We calculated the maximum absolute value of each parameter for each participant, resulting in six parameters. We used all six parameters, the maximum of the six parameters, the maximum of the three translation parameters, the maximum of the three rotation parameters, the sum of the six parameters, the sum of the three translation parameters, and the sum of the three rotation parameters to conduct correlation analyses. Framewise displacement reflected frame-to-frame head motion patterns. We used mean framewise displacement to conduct correlation analyses.

### Leave-Two-Out Prediction

We used the leave-two-out cross-validation method to examine the accuracy of the predictive effects. In each iteration, two participants were excluded as the test set, and the remaining participants composed the training set. Because each pair of participants was excluded once, except for the pairs with the same life satisfaction change score, there were 1,548 iterations. In the prediction stage, we obtained a predicted life satisfaction change score in each model for each of the two participants in the test set. The two predicted life satisfaction change scores and the two observed scores were compared. The accuracy of the iteration was 1 when the results of the two comparisons were consistent; otherwise, the accuracy was 0. In the model evaluation stage, the mean accuracy of all iterations was defined as the predictive power. The other procedures were similar to those of the leave-one-out prediction. We compared the leave-two-out prediction results with a random binomial distribution to test the effectiveness of the prediction (see the Supplementary Results and Supplementary Figure 1).

## Results

### Whole-Brain Prediction

**FIGURE 2:**
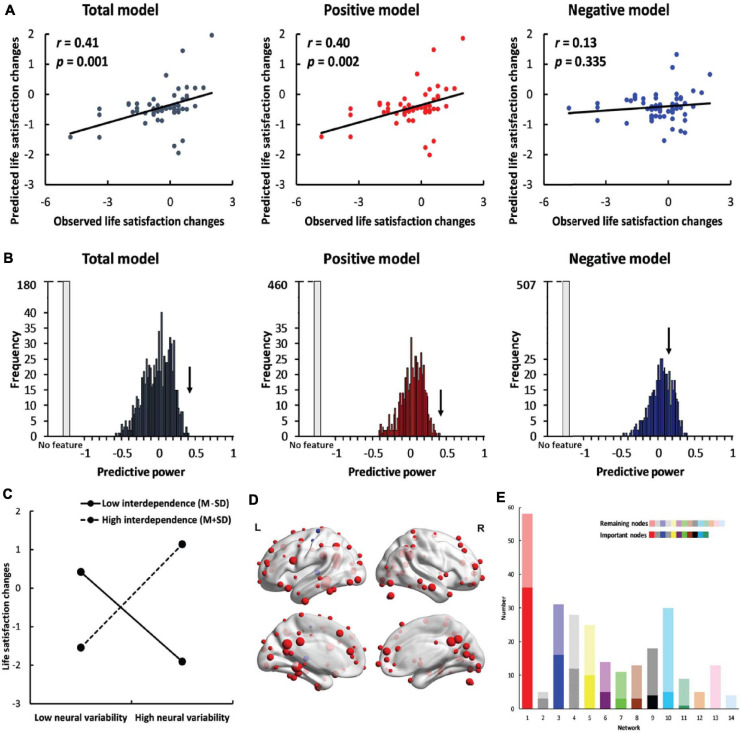
Results of whole-brain prediction. **(A)** The predictive power (correlation between predicted life satisfaction change scores and observed life satisfaction change scores) of the total model (*left*, dark gray), positive model (*middle*, red) and negative model (*right*, blue). **(B)** The results of permutation tests in the total model (*left*, dark gray), positive model (*middle*, red) and negative model (*right*, blue). The black arrow indicates the observed predictive power in the three models. The light gray bar indicates the permutations in which no feature was selected in at least one iteration. **(C)** The simple effects of whole-brain prediction. **(D)** The location of important nodes (the nodes that were selected as features in more than 95% of the iterations) in the positive feature set (red) and in the negative feature set (blue). **(E)** The distribution of important nodes in the default mode network (red), memory retrieval network (gray), visual network (blue), uncertain network (striated), frontoparietal task control network (yellow), cingulo-opercular task control network (purple), dorsal attention network (green), subcortical network (brown), salience network (black), hand sensory-somatomotor network (cyan), ventral attention network (teal), mouth sensory-somatomotor network (orange), auditory network (pink) and cerebellar network (pale blue). The dark colors indicate the important nodes, and the light colors indicate the remaining nodes. The networks were ranked by the percentage of the number of important nodes out of all nodes in each specific network.

Self-dependent neural variability successfully predicted life satisfaction change scores in the total model (*r* = 0.41, *p* = 0.001, Figure 2A) and in the positive model (*r* = 0.40, *p* = 0.002). However, SDNV failed to predict life satisfaction change scores in the negative model (*r* = 0.13, *p* = 0.335). The results indicate that self-construal moderated the relationship between neural variability and life satisfaction changes. The results were robust across manipulations of feature selection thresholds (∝ = 0.10, 0.05, 0.01) and window lengths (window length = 30, 40, and 50 s) (Supplementary Figures 2–4 and the Supplementary Results).

To control for the effects of neural variability and interdependent self-construal, we added the two variables as regressors in the feature selection stage and in the model establishment stage. SDNV remained predictive of life satisfaction change scores in the total model (*r* = 0.38, *p* = 0.003) and in the positive model (*r* = 0.37, *p* = 0.004), whereas the predictive effect was still not significant in the negative model (*r* = 0.08, *p* = 0.538). The results suggest that the predictive effects were robust after controlling for neural variability and interdependent self-construal.

To further confirm the significance of the predictive effects, we performed permutation tests. In the total model and the positive model, the observed predictive power significantly differed from the predictive power in the null distribution (total model: *p* < 0.001; positive model: *p* < 0.001, Figure 2B). In the negative model, the observed predictive power did not significantly differ from the predictive power in the null distribution (*p* = 0.138).

We conducted a simple effects analysis using the mean neural variability of important nodes to examine the relationship between neural variability and life satisfaction change scores in individuals with high interdependent self-construal (M+SD) and in those with low interdependent self-construal (M-SD). Neural variability was positively correlated with life satisfaction change scores [*b* = 1.40, SE = 0.42, *p* = 0.002, 95% CI = (0.56, 2.24), Figure 2C] in interdependent individuals (mean = 1.05) but negatively correlated with life satisfaction change scores [*b* = −1.14, SE = 0.31, *p* < 0.001, 95% CI = (−1.77, −0.51)] in independent individuals (mean = −0.98). The results indicate that interdependent individuals with higher neural variability and independent individuals with lower neural variability became more satisfied with their lives. We also conducted a simple effects analysis using the node whose SDNV was most correlated with life satisfaction change scores and found the same patterns of results (Supplementary Figure 5 and the Supplementary Results).

### Network-Based Prediction

In the whole-brain prediction, there were 101 important nodes in total, among which 98 nodes were included in the positive feature set and three nodes were included in the negative feature set (Figure 2D). The important nodes were mainly distributed in the default mode network (*n* = 36), visual network (*n* = 16) and frontoparietal task control network (*n* = 10) (Figure 2E). We also calculated the percentage of the important nodes in each of the 14 networks. The percentage of important nodes was the highest in the default mode network (62.1%, Figure 2E), suggesting the important role of this network in predicting individuals’ life satisfaction change scores.

**FIGURE 3:**
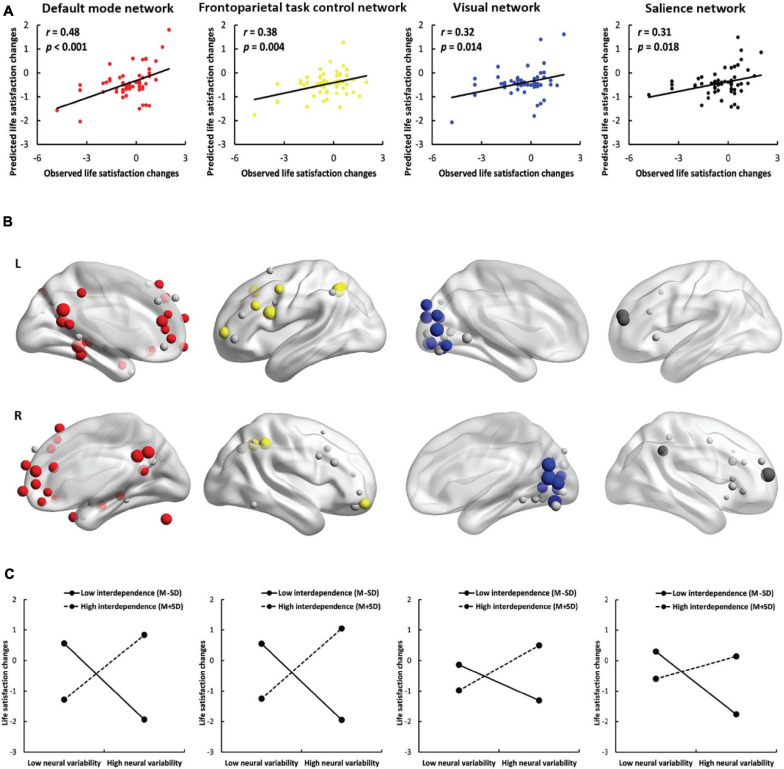
Results of network-based prediction. **(A)** The predictive power of the positive model in the default mode network (red), frontoparietal task control network (yellow), visual network (blue) and salience network (black). **(B)** The location of important nodes in the default mode network (red), frontoparietal task control network (yellow), visual network (blue), salience network (black) and the remaining nodes (gray) in the four networks. **(C)** The simple effects of the network-based prediction in the four networks.

To study the contribution of each network (except for the uncertain network), we used the nodes within the network instead of the nodes across the whole brain to perform the prediction analyses. SDNV significantly predicted life satisfaction change scores in the default mode network (*r* = 0.48, *p* < 0.001, Figures 3A,B), frontoparietal task control network (*r* = 0.38, *p* = 0.004), visual network (*r* = 0.32, *p* = 0.014) and salience network (*r* = 0.31, *p* = 0.018) in the positive model. Except for the total model of the salience network (*r* = 0.28, *p* = 0.033), in the total model or in the negative model, no feature was selected in at least one iteration, and therefore, prediction analysis was not performed. The predictive effects of the remaining networks did not survive false discovery rate (FDR) correction (Supplementary Table 1). These results further confirmed that the default mode, frontoparietal task control, visual, and salience networks contributed to the prediction. The results of the default mode network were robust across feature selection thresholds (see the Supplementary Results).

Neural variability within the default mode network, frontoparietal task control network, visual network, and salience network was positively correlated with life satisfaction change scores in interdependent individuals [default mode network: *b* = 1.12, SE = 0.35, *p* = 0.002, 95% CI = (0.42, 1.82); frontoparietal task control network: *b* = 1.21, SE = 0.39, *p* = 0.003, 95% CI = (0.43, 1.99); visual network: *b* = 0.77, SE = 0.31, *p* = 0.015, 95% CI = (0.15, 1.39); salience network: *b* = 0.40, SE = 0.30, *p* = 0.180, 95% CI = (−0.19, 0.99)] but negatively correlated with life satisfaction change scores in independent individuals [default mode network: *b* = −1.23, SE = 0.28, *p* < 0.001, 95% CI = (−1.79, −0.67); frontoparietal task control network: *b* = −1.23, SE = 0.29, *p* < 0.001, 95% CI = (−1.81, −0.65); visual network: *b* = −0.57, SE = 0.24, *p* = 0.019, 95% CI = (−1.05, −0.10); salience network: *b* = −1.02, SE = 0.25, *p* < 0.001, 95% CI = (−1.52, −0.52); Figure 3C]. The results show that interdependent individuals with higher neural variability and independent individuals with lower neural variability in these four networks became more satisfied with their lives. The implications of network-based prediction further confirmed the implications of whole-brain prediction. We also conducted a simple effects analysis using the node whose SDNV was most correlated with life satisfaction change scores and found the same patterns of results (Supplementary Figure 6 and the Supplementary Results).

### Meta-Analytic Decoding

To understand the structural location and psychological function related to the important nodes in the whole-brain prediction and in the network-based prediction, we decoded the map of the important nodes or the remaining nodes in the two prediction analyses using the Neurosynth Image Decoder (Yarkoni et al., 2011).

**FIGURE 4:**
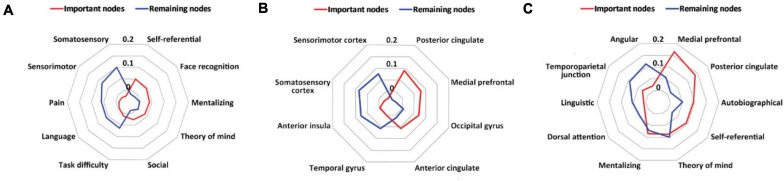
Results of meta-analytic decoding. **(A)** The psychological constructs related to the important nodes (red) and the remaining nodes (blue) in the whole-brain prediction. **(B)** The brain structures related to the important nodes (red) and the remaining nodes (blue) in the whole-brain prediction. **(C)** The psychological constructs and brain structures related to the important nodes (red) and the remaining nodes (blue) in the default mode network-based prediction.

In the whole-brain prediction, the important nodes were more related to psychological constructs associated with the social function than the remaining nodes, including self-referential, face recognition, mentalizing, theory of mind and social, whereas the remaining nodes were more related to psychological constructs associated with the sensory and cognitive functions, including somatosensory, sensorimotor, task difficulty, pain and language (Figure 4A). In addition, the important nodes were mainly located in the posterior cingulate, medial prefrontal, occipital gyrus and anterior cingulate, whereas the remaining nodes were mainly located in the somatosensory cortex, sensorimotor cortex, anterior insula, and temporal gyrus (Figure 4B).

In the default mode network-based prediction, the important nodes were more related to psychological constructs and brain structures associated with social and self-oriented functions and structures, including autobiographical, self-referential, theory of mind, the medial prefrontal, and the posterior cingulate, whereas the remaining nodes were more related to psychological constructs and brain structures associated with cognitive and other-oriented functions and structures, including linguistic, dorsal attention, angular and the temporoparietal junction (Figure 4C).

### Specificity of the Predictive Effects

To test whether the predictive effects were specific for SDNV, we used neural variability or interdependent self-construal to perform the prediction analyses or Pearson correlation analysis, respectively. Neural variability failed to predict life satisfaction change scores in the total model (*r* = −0.46, *p* < 0.001), positive model (*r* = −0.36, *p* = 0.005) and negative model (*r* = −0.26, *p* = 0.048). Interdependent self-construal was not significantly correlated with life satisfaction change scores (*r* = 0.13, *p* = 0.314). The results illustrate that neither neural variability nor self-construal could predict life satisfaction change scores.

To test whether the predictive effects were specific for the life satisfaction change scores, we used the life satisfaction score at the first assessment or the life satisfaction score at the second assessment to perform the prediction analyses. SDNV failed to predict the life satisfaction score at the first assessment (total model: *r* = 0.10, *p* = 0.458; positive model: *r* = 0.21, *p* = 0.102; negative model: *r* = 0.09, *p* = 0.520) and at the second assessment (total model: *r* = −0.06, *p* = 0.658; positive model: *r* = −0.07, *p* = 0.622; negative model: *r* = 0.11, *p* = 0.415).

We also tested whether SDNV could predict individuals’ place of origin (urban–rural, independent objective information) or ethnic identity (independent subjective values), which were independent of life satisfaction change scores. SDNV failed to predict individuals’ place of origin (total model: *r* = −0.25, *p* = 0.060; positive model: *r* = −0.12, *p* = 0.367) or ethnic identity (total model: *r* = 0.02, *p* = 0.850; positive model: *r* = 0.02, *p* = 0.855). In the negative model of the two prediction analyses, no feature was selected in at least one iteration, so the prediction analyses were not performed. The results illustrate that the predictive effects were specific for life satisfaction change scores.

To exclude the effects of head motion, we conducted Pearson correlation analyses between the head motion parameters (six rigid-body parameters and framewise displacement) and life satisfaction change scores. Neither the six rigid-body parameters nor framewise displacement were significantly correlated with life satisfaction change scores (Supplementary Table 2). The results illustrate that the head motion patterns were not predictive of life satisfaction change scores; thus, the confounding effects of head motion were excluded.

## Discussion

In the current research, we examine the hypothesis that self-construal moderates the relationship between neural variability and life satisfaction changes in university freshmen. Interdependent individuals with higher neural variability and independent individuals with lower neural variability become more satisfied with their lives. The default mode network contributes to the prediction. The predictive effects are robust (i.e., across window lengths and feature selection thresholds) and generalize to novel individuals.

We find that SDNV predicts life satisfaction changes. The results demonstrate that SDNV is an effective indicator of subjective well-being. Previous brain imaging studies on subjective well-being have been based on traditional static indicators, including neural activation (Urry et al., 2004; Kong et al., 2015c) and functional connectivity (Li et al., 2020; Luo et al., 2016), ignoring the dynamic nature of neural activities. In the current research, neural variability, the temporal variability of regional functional architecture (Zhang et al., 2016), is used to reveal the neural mechanism of subjective well-being from a dynamic perspective. In addition, the current research shows that neural variability can predict not only mental disorders (Hou et al., 2018; Long et al., 2020; Zhang et al., 2016) but also positive changes in mental health (i.e., life satisfaction changes), which expands relevant studies to a new domain.

Specifically, interdependent individuals with higher neural variability and independent individuals with lower neural variability become more satisfied with their lives. The results support the culture–behavior–brain (CBB) loop model proposed by Han and Ma (2015). According to the CBB loop, the social environment shapes the brain via behavior, and in turn, the brain leads behavior to adapt to the social environment. In other words, the social environment and the brain interact to influence behavior. University freshmen experience great changes in their social environment, including their place of residence and interpersonal relationships (Boujut and Bruchon-Schweitzer, 2009). Adaptation to the new social environment is vital to subjective well-being (Bailey and Phillips, 2016; Zhou and Lin, 2016). Self-construal reflects the response to the social environment of individuals (Markus and Kitayama, 1991). Interdependent individuals emphasize flexibility across different social contexts (Cheng et al., 2011), whereas independent individuals value self-consistency (Suh, 2002). When interdependent individuals have a flexible brain (i.e., high neural variability) or independent individuals have a stable brain (i.e., low neural variability), their social response matches the functional organization of their brain, which enables them to spend less time and effort adapting to the new social environment, which in turn increases their life satisfaction. In the current research, we find that self-construal moderates the relationship between neural variability and life satisfaction changes in Chinese university freshmen. Future studies may test whether the predictive effects can generalize to other social environments, such as new jobs or other cultures.

Moreover, the default mode network contributes to the prediction of life satisfaction changes. The default mode network is involved in social processes and overlaps the social network in structures and functions (Rilling et al., 2008; Mars et al., 2012; Li et al., 2014). The default mode network shows neural activation and within-network functional connectivity during autobiographical memory, prospection and theory of mind tasks (Spreng and Grady, 2010). Thus, the default mode network is involved in reflecting the past and the future of oneself and mentalizing the mind of others. It provides individuals with the neural and psychological basis to evaluate their present life situations and to compare with others, which affects subjective well-being (Hagerty, 2000). The results of our meta-analytic decoding based on whole-brain prediction support the important role of social processes in the prediction of life satisfaction changes. In addition, the results of our meta-analytic decoding based on default mode network prediction show that self-oriented functions and structures are related to the prediction, specifically the medial prefrontal cortex and posterior cingulate cortex. The medial prefrontal cortex and posterior cingulate cortex are involved in various domains (e.g., memory, emotional, and social) of self-referential processes (Northoff et al., 2006). The neural patterns of the two regions distinguish the self from others and differentiate various dimensions (i.e., mental, physical, and social) of self-knowledge (Feng et al., 2018). Specifically, the medial prefrontal cortex is involved in the representation and evaluation of self-referential information, whereas the posterior cingulate cortex is involved in the integration of self-referential information in the personal context (Northoff and Bermpohl, 2004) and provides further support for the general self-evaluation of life satisfaction. Our results demonstrate that social processes, especially self-oriented processes, play an important role in the prediction of life satisfaction changes.

The current study examines the predictive effects in Chinese culture, where interdependent self-construal is dominant and adaptive (Markus and Kitayama, 1991). A previous study has shown that the relationships between self-construal and life satisfaction vary with culture (Cheng et al., 2011). Future studies may test whether the predictive effects can generalize to other cultures, including Western cultures, where independent self-construal is dominant. Furthermore, university freshmen are chosen as the sample since they are experiencing an important transition under a highly variable environment, and future studies may test whether the predictive effects can be applied to a less variable environment. In addition, the Satisfaction with Life Scale (Diener et al., 1985) is used to measure life satisfaction, which assesses overall life satisfaction. Future studies may test whether the predictive effects can be applied to different domains (i.e., finance, friendship, and health) of life satisfaction using various assessments (Michalos, 1980). Finally, the current study examines the predictive effects on changes in life satisfaction. Given that neural variability is an effective indicator of various mental disorders (Hou et al., 2018; Long et al., 2020; Zhang et al., 2016), future studies should test whether neural variability can predict the recovery or deterioration of mental disorders in a clinical sample (i.e., patients with major depressive disorder, schizophrenia, and attention deficit hyperactivity disorder) or changes in negative mood in a subclinical sample.

In conclusion, SDNV predicts life satisfaction changes in university freshmen. Interdependent individuals with higher neural variability and independent individuals with lower neural variability become more satisfied with their lives. The current research sheds light on the neural mechanism of life satisfaction from a dynamic perspective and complements previous findings. Furthermore, the current research has important implications for gaining deeper insight into the adaptation and subjective well-being of university freshmen.

## Data Availability Statement

The original contributions presented in the study are included in the article/Supplementary Material, further inquiries can be directed to the corresponding author.

## Ethics Statement

The studies involving human participants were reviewed and approved by Department of Psychology of Sun Yat-sen University Ethics Committee. The patients/participants provided their written informed consent to participate in this study.

## Author Contributions

SL designed the research. LF and SL collected and analyzed the data and wrote the manuscript. All authors commented on the manuscript.

## Conflict of Interest

The authors declare that the research was conducted in the absence of any commercial or financial relationships that could be construed as a potential conflict of interest.

## References

[B1] Allen E. A. Damaraju E. Plis S. M. Erhardt E. B. Eichele T. Calhoun V. D. (2014). Tracking whole-brain connectivity dynamics in the resting state. *Cereb. Cortex* 24 663–676. 10.1093/cercor/bhs352 23146964 PMC3920766

[B2] Andrews F. M. Withey S. B. (1976). *Social Indicators of Well-Being: America’s Perception of Life Quality.* New York, NY: Plenum.

[B3] Bailey T. H. Phillips L. J. (2016). The influence of motivation and adaptation on students’ subjective well-being, meaning in life and academic performance. *High. Educ. Res. Dev.* 35 201–216. 10.1080/07294360.2015.1087474

[B4] Bassett D. S. Sporns O. (2017). Network neuroscience. *Nat. Neurosci.* 20 353–364. 28230844 10.1038/nn.4502 PMC5485642

[B5] Biswal B. Zerrin Yetkin F. Haughton V. M. Hyde J. S. (1995). Functional connectivity in the motor cortex of resting human brain using echo-planar MRI. *Magn. Reson. Med.* 34 537–541. 10.1002/mrm.1910340409 8524021

[B6] Boujut E. Bruchon-Schweitzer M. (2009). A construction and validation of a freshman stress questionnaire: an exploratory study. *Psychol. Rep.* 104 680–692. 10.2466/pr0.104.2.680-692 19610500

[B7] Chang C. Glover G. H. (2010). Time–frequency dynamics of resting-state brain connectivity measured with fMRI. *NeuroImage* 50 81–98. 10.1016/j.neuroimage.2009.12.011 20006716 PMC2827259

[B8] Chen H. Liu K. Zhang B. Zhang J. Xue X. Lin Y. (2019). More optimal but less regulated dorsal and ventral visual networks in patients with major depressive disorder. *J. Psychiatr. Res.* 110 172–178. 10.1016/j.jpsychires.2019.01.005 30654314

[B9] Cheng C. Wang F. Golden D. L. (2011). Unpacking cultural differences in interpersonal flexibility: role of culture-related personality and situational factors. *J. Cross Cult. Psychol.* 42 425–444. 10.1177/0022022110362755

[B10] Choe A. S. Nebel M. B. Barber A. D. Cohen J. R. Xu Y. Pekar J. J. (2017). Comparing test-retest reliability of dynamic functional connectivity methods. *NeuroImage* 158 155–175. 10.1016/j.neuroimage.2017.07.005 28687517 PMC5614828

[B11] Cui Z. Gong G. (2018). The effect of machine learning regression algorithms and sample size on individualized behavioral prediction with functional connectivity features. *NeuroImage* 178 622–637. 10.1016/j.neuroimage.2018.06.001 29870817

[B12] Dayan E. Cohen L. G. (2011). Neuroplasticity subserving motor skill learning. *Neuron* 72 443–454. 10.1016/j.neuron.2011.10.008 22078504 PMC3217208

[B13] Deng Y. Liu K. Cheng D. Zhang J. Chen H. Chen B. (2019). Ventral and dorsal visual pathways exhibit abnormalities of static and dynamic connectivities, respectively, in patients with schizophrenia. *Schizophrenia Res.* 206 103–110. 10.1016/j.schres.2018.12.005 30545760

[B14] Diener E. D. Seligman M. E. P. (2002). Very happy people. *Psychol. Sci.* 13 81–84. 10.1111/1467-9280.00415 11894851

[B15] Diener E. D. Emmons R. A. Larsen R. J. Griffin S. (1985). The satisfaction with life scale. *J. Pers. Assess.* 49 71–75. 16367493 10.1207/s15327752jpa4901_13

[B16] Dong D. Duan M. Wang Y. Zhang X. Jia X. Li Y. (2019). Reconfiguration of dynamic functional connectivity in sensory and perceptual system in schizophrenia. *Cereb. Cortex* 29 3577–3589. 10.1093/cercor/bhy232 30272139

[B17] Erdogan B. Bauer T. N. Truxillo D. M. Mansfield L. R. (2012). Whistle while you work: a review of the life satisfaction literature. *J. Manag.* 38 1038–1083. 10.1177/0149206311429379

[B18] Feng C. Yan X. Huang W. Han S. Ma Y. (2018). Neural representations of the multidimensional self in the cortical midline structures. *NeuroImage* 183 291–299. 10.1016/j.neuroimage.2018.08.018 30118871

[B19] Guney Y. (2009). Exogenous and endogenous factors influencing students’ performance in undergraduate accounting modules. *Acc. Educ.* 18 51–73. 10.1080/09639280701740142

[B20] Guo S. Zhao W. Tao H. Liu Z. Palaniyappan L. (2018). The instability of functional connectivity in patients with schizophrenia and their siblings: a dynamic connectivity study. *Schizophrenia Res.* 195 183–189. 10.1016/j.schres.2017.09.035 29153446

[B21] Hagerty M. R. (2000). Social comparisons of income in one’s community: evidence from national surveys of income and happiness. *J. Pers. Soc. Psychol.* 78 764–771. 10.1037/0022-3514.78.4.764 10794379

[B22] Han S. Humphreys G. (2016). Self-construal: a cultural framework for brain function. *Curr. Opin. Psychol.* 8 10–14. 10.1016/j.copsyc.2015.09.013 29506783

[B23] Han S. Ma Y. (2015). A culture–behavior–brain loop model of human development. *Trends Cogn. Sci.* 19 666–676. 10.1016/j.tics.2015.08.010 26440111

[B24] Headey B. Kelley J. Wearing A. (1993). Dimensions of mental health: life satisfaction, positive affect, anxiety and depression. *Soc. Indic. Res.* 29 63–82. 10.1007/bf01136197

[B25] Heintzelman S. J. Bacon P. L. (2015). Relational self-construal moderates the effect of social support on life satisfaction. *Pers. Individ. Dif.* 73 72–77. 10.1016/j.paid.2014.09.021

[B26] Hope N. Koestner R. Milyavskaya M. (2014). The role of self-compassion in goal pursuit and well-being among university freshmen. *Self Identity* 13 579–593. 10.1080/15298868.2014.889032

[B27] Hou Z. Kong Y. He X. Yin Y. Zhang Y. Yuan Y. (2018). Increased temporal variability of striatum region facilitating the early antidepressant response in patients with major depressive disorder. *Progress Neuropsychopharmacol. Biol. Psychiatry* 85 39–45. 10.1016/j.pnpbp.2018.03.026 29608926

[B28] Hutchison R. M. Womelsdorf T. Allen E. A. Bandettini P. A. Calhoun V. D. Corbetta M. (2013). Dynamic functional connectivity: promise, issues, and interpretations. *NeuroImage* 80 360–378. 10.1016/j.neuroimage.2013.05.079 23707587 PMC3807588

[B29] Jiang C. Varnum M. E. Hou Y. Han S. (2014). Distinct effects of self-construal priming on empathic neural responses in Chinese and Westerners. *Soc. Neurosci.* 9 130–138. 10.1080/17470919.2013.867899 24341541

[B30] Kalivas P. W. O’Brien C. (2008). Drug addiction as a pathology of staged neuroplasticity. *Neuropsychopharmacology* 33 166–180. 10.1038/sj.npp.1301564 17805308

[B31] Kemmelmeier M. Oyserman D. (2001). The ups and downs of thinking about a successful other: self-construals and the consequences of social comparisons. *Eur. J. Soc. Psychol.* 31 311–320. 10.1002/ejsp.47

[B32] Kim E. J. Kyeong S. Cho S. W. Chun J. W. Park H. J. Kim J. (2016). Happier people show greater neural connectivity during negative self-referential processing. *PLoS One* 11:e0149554. 10.1371/journal.pone.0149554 26900857 PMC4763307

[B33] Kong F. Ding K. Zhao J. (2015b). The relationships among gratitude, self-esteem, social support and life satisfaction among undergraduate students. *J. Happiness Stud.* 16 477–489. 10.1007/s10902-014-9519-2

[B34] Kong F. Ding K. Yang Z. Dang X. Hu S. Song Y. (2015a). Examining gray matter structures associated with individual differences in global life satisfaction in a large sample of young adults. *Soc. Cogn. Affect. Neurosci.* 10 952–960. 10.1093/scan/nsu144 25406366 PMC4483566

[B35] Kong F. Hu S. Wang X. Song Y. Liu J. (2015c). Neural correlates of the happy life: the amplitude of spontaneous low frequency fluctuations predicts subjective well-being. *NeuroImage* 107 136–145. 10.1016/j.neuroimage.2014.11.033 25463465

[B36] Lewis D. A. González-Burgos G. (2008). Neuroplasticity of neocortical circuits in schizophrenia. *Neuropsychopharmacology* 33 141–165. 10.1038/sj.npp.1301563 17805309

[B37] Li R. Zhu X. Zheng Z. Wang P. Li J. (2020). Subjective well-being is associated with the functional connectivity network of the dorsal anterior insula. *Neuropsychologia* 141:107393. 10.1016/j.neuropsychologia.2020.107393 32057936

[B38] Li W. Mai X. Liu C. (2014). The default mode network and social understanding of others: what do brain connectivity studies tell us. *Front. Hum. Neurosci.* 8:74. 10.3389/fnhum.2014.00074 24605094 PMC3932552

[B39] Liao X. Yuan L. Zhao T. Dai Z. Shu N. Xia M. (2015). Spontaneous functional network dynamics and associated structural substrates in the human brain. *Front. Hum. Neurosci.* 9:478. 10.3389/fnhum.2015.00478 26388757 PMC4559598

[B40] Lim C. Putnam R. D. (2010). Religion, social networks, and life satisfaction. *Am. Sociol. Rev.* 75 914–933. 10.1177/0003122410386686

[B41] Long Y. Liu Z. Chan C. K. Y. Wu G. Xue Z. Pan Y. (2020). Altered temporal variability of local and large-scale resting-state brain functional connectivity patterns in schizophrenia and bipolar disorder. *Front. Psychiatry* 11:422. 10.3389/fpsyt.2020.00422 32477194 PMC7235354

[B42] Luo S. Ma Y. Liu Y. Li B. Wang C. Shi Z. (2015). Interaction between oxytocin receptor polymorphism and interdependent culture values on human empathy. *Soc. Cogn. Affect. Neurosci.* 10 1273–1281. 10.1093/scan/nsv019 25680993 PMC4560951

[B43] Luo Y. Kong F. Qi S. You X. Huang X. (2016). Resting-state functional connectivity of the default mode network associated with happiness. *Soc. Cogn. Affect. Neurosci.* 11 516–524. 26500289 10.1093/scan/nsv132 PMC4769634

[B44] Ma Y. Bang D. Wang C. Allen M. Frith C. Roepstorff A. (2014). Sociocultural patterning of neural activity during self-reflection. *Soc. Cogn. Affect. Neurosci.* 9 73–80. 10.1093/scan/nss103 22956678 PMC3871729

[B45] Markus H. R. Kitayama S. (1991). Culture and the self: Implications for cognition, emotion, and motivation. *Psychol. Rev.* 98 224–253. 10.1037/0033-295x.98.2.224

[B46] Mars R. B. Neubert F. X. Noonan M. P. Sallet J. Toni I. Rushworth M. F. (2012). On the relationship between the “default mode network” and the “social brain”. *Front. Hum. Neurosci.* 6:189. 10.3389/fnhum.2012.00189 22737119 PMC3380415

[B47] Michalos A. C. (1980). Satisfaction and happiness. *Soc. Indic. Res.* 8 385–422.

[B48] Mroczek D. K. Spiro A. III (2005). Change in life satisfaction during adulthood: findings from the veterans affairs normative aging study. *J. Pers. Soc. Psychol.* 88 189–202. 10.1037/0022-3514.88.1.189 15631584

[B49] Münte T. F. Altenmüller E. Jäncke L. (2002). The musician’s brain as a model of neuroplasticity. *Nat. Rev. Neurosci.* 3 473–478. 12042882 10.1038/nrn843

[B50] Northoff G. Bermpohl F. (2004). Cortical midline structures and the self. *Trends Cogn. Sci.* 8 102–107. 10.1016/j.tics.2004.01.004 15301749

[B51] Northoff G. Heinzel A. De Greck M. Bermpohl F. Dobrowolny H. Panksepp J. (2006). Self-referential processing in our brain—a meta-analysis of imaging studies on the self. *NeuroImage* 31 440–457. 10.1016/j.neuroimage.2005.12.002 16466680

[B52] Phinney J. S. (1992). The multigroup ethnic identity measure: a new scale for use with diverse groups. *J. Adolesc. Res.* 7 156–176. 10.1177/074355489272003

[B53] Pittenger C. Duman R. S. (2008). Stress, depression, and neuroplasticity: a convergence of mechanisms. *Neuropsychopharmacology* 33 88–109. 10.1038/sj.npp.1301574 17851537

[B54] Power J. D. Cohen A. L. Nelson S. M. Wig G. S. Barnes K. A. Church J. A. (2011). Functional network organization of the human brain. *Neuron* 72 665–678. 22099467 10.1016/j.neuron.2011.09.006 PMC3222858

[B55] Preti M. G. Bolton T. A. Van De Ville D. (2017). The dynamic functional connectome: state-of-the-art and perspectives. *NeuroImage* 160 41–54. 10.1016/j.neuroimage.2016.12.061 28034766

[B56] Qin P. Northoff G. (2011). How is our self related to midline regions and the default-mode network? *NeuroImage* 57 1221–1233. 10.1016/j.neuroimage.2011.05.028 21609772

[B57] Raichle M. E. MacLeod A. M. Snyder A. Z. Powers W. J. Gusnard D. A. Shulman G. L. (2001). A default mode of brain function. *Proc. Natl. Acad. Sci. U.S.A.* 98 676–682. 11209064 10.1073/pnas.98.2.676 PMC14647

[B58] Ray R. D. Shelton A. L. Hollon N. G. Matsumoto D. Frankel C. B. Gross J. J. (2010). Interdependent self-construal and neural representations of self and mother. *Soc. Cogn. Affect. Neurosci.* 5 318–323. 10.1093/scan/nsp039 19822601 PMC2894675

[B59] Rilling J. K. Dagenais J. E. Goldsmith D. R. Glenn A. L. Pagnoni G. (2008). Social cognitive neural networks during in-group and out-group interactions. *NeuroImage* 41 1447–1461. 10.1016/j.neuroimage.2008.03.044 18486491

[B60] Sheng J. Shen Y. Qin Y. Zhang L. Jiang B. Li Y. (2018). Spatiotemporal, metabolic, and therapeutic characterization of altered functional connectivity in major depressive disorder. *Hum. Brain Mapp.* 39 1957–1971. 10.1002/hbm.23976 29341320 PMC6866283

[B61] Shin D. C. Johnson D. M. (1978). Avowed happiness as an overall assessment of the quality of life. *Soc. Indic. Res.* 5 475–492. 10.1007/bf00352944

[B62] Singelis T. M. (1994). The measurement of independent and interdependent self-construals. *Pers. Soc. Psychol. Bull.* 20 580–591. 10.1177/0146167294205014

[B63] Spreng R. N. Grady C. L. (2010). Patterns of brain activity supporting autobiographical memory, prospection, and theory of mind, and their relationship to the default mode network. *J. Cogn. Neurosci.* 22 1112–1123. 10.1162/jocn.2009.21282 19580387

[B64] Spreng R. N. Mar R. A. Kim A. S. (2009). The common neural basis of autobiographical memory, prospection, navigation, theory of mind, and the default mode: a quantitative meta-analysis. *J. Cogn. Neurosci.* 21 489–510. 10.1162/jocn.2008.21029 18510452

[B65] Stapel D. A. Koomen W. (2001). I, we, and the effects of others on me: how self-construal level moderates social comparison effects. *J. Pers. Soc. Psychol.* 80 766–781. 10.1037/0022-3514.80.5.766 11374748

[B66] Suh E. M. (2002). Culture, identity consistency, and subjective well-being. *J. Pers. Soc. Psychol.* 83 1378–1391. 10.1037/0022-3514.83.6.1378 12500819

[B67] Urry H. L. Nitschke J. B. Dolski I. Jackson D. C. Dalton K. M. Mueller C. J. (2004). Making a life worth living: neural correlates of well-being. *Psychol. Sci.* 15 367–372. 10.1111/j.0956-7976.2004.00686.x 15147488

[B68] Wang C. Oyserman D. Liu Q. Li H. Han S. (2013). Accessible cultural mind-set modulates default mode activity: evidence for the culturally situated brain. *Soc. Neurosci.* 8 203–216. 10.1080/17470919.2013.775966 23485156

[B69] Wang Q. (2001). Culture effects on adults’ earliest childhood recollection and self-description: implications for the relation between memory and the self. *J. Pers. Soc. Psychol.* 81 220–233. 10.1037/0022-3514.81.2.220 11519928

[B70] White K. Lehman D. R. Cohen D. (2006). Culture, self-construal, and affective reactions to successful and unsuccessful others. *J. Exp. Soc. Psychol.* 42 582–592. 10.1016/j.jesp.2005.10.001

[B71] Yan C. Zang Y. (2010). DPARSF: a MATLAB toolbox for” pipeline” data analysis of resting-state fMRI. *Front. Syst. Neurosci.* 4:13. 10.3389/fnsys.2010.00013 20577591 PMC2889691

[B72] Yarkoni T. Poldrack R. A. Nichols T. E. Van Essen D. C. Wager T. D. (2011). Large-scale automated synthesis of human functional neuroimaging data. *Nat. Methods* 8 665–670. 10.1038/nmeth.1635 21706013 PMC3146590

[B73] Zalesky A. Fornito A. Cocchi L. Gollo L. L. Breakspear M. (2014). Time-resolved resting-state brain networks. *Proc. Natl. Acad. Sci. U.S.A.* 111 10341–10346. 24982140 10.1073/pnas.1400181111 PMC4104861

[B74] Zhang J. Cheng W. Liu Z. Zhang K. Lei X. Yao Y. (2016). Neural, electrophysiological and anatomical basis of brain-network variability and its characteristic changes in mental disorders. *Brain* 139 2307–2321. 10.1093/brain/aww143 27421791

[B75] Zhang W. Li S. Wang X. Gong Y. Yao L. Xiao Y. (2018). Abnormal dynamic functional connectivity between speech and auditory areas in schizophrenia patients with auditory hallucinations. *NeuroImage* 19 918–924. 10.1016/j.nicl.2018.06.018 30003029 PMC6039841

[B76] Zhou M. Lin W. (2016). Adaptability and life satisfaction: the moderating role of social support. *Front. Psychol.* 7:1134. 10.3389/fpsyg.2016.01134 27516753 PMC4963457

[B77] Zou H. Yang J. (2019). Temporal variability–based functional brain lateralization study in ADHD. *J. Attent. Disord.* 25 839–847. 10.1177/1087054719859074 31268386

[B78] Zou X. Ingram P. Higgins E. T. (2015). Social networks and life satisfaction: The interplay of network density and regulatory focus. *Motiv. Emot.* 39 693–713. 10.1007/s11031-015-9490-1 26380535 PMC4565878

