# Supplementary material for: Symphony of Well-Being: Harmony Between Neural Variability and Self-Construal
Source: Front Hum Neurosci. 2021 Jun 30;15:679086. doi: 10.3389/fnhum.2021.679086 (PMC8278332; doi:10.3389/fnhum.2021.679086)
Supplement: Supplementary file 1 [file Data_Sheet_1.docx]

**SUPPLEMENTARY MATERIALS**

**Symphony of Well-being: Harmony Between Neural Variability and Self-Construal**

**Supplementary results**

Figure S1-S6

Table S1-S2

**RESULTS**

**Leave-two-out prediction**

We used the leave-two-out cross-validation method to examine the accuracy of the prediction effects. In the total model, the mean accuracy was 1035/1548 (66.9%, Figure S1). In the positive model, the mean accuracy was 1032/1548 (66.7%). In the negative model, the mean accuracy was 850/1548 (54.9%). Moreover, we compared the leave-two-out prediction results with a random binomial distribution to test the effectiveness of the prediction. The mean accuracy of the prediction effects in the three models were significant (total model: *z* = 13.27, *p* < 0.001; positive model: *z* = 13.11, *p* < 0.001; negative model: *z* = 3.86, *p* < 0.001).

The prediction was more accurate in the iterations in which the difference of life satisfaction change scores between the two participants in the test set was greater. In the iterations in which the difference was greater than 1 SD of life satisfaction change scores, the mean accuracy was 512/661 (77.5%, *z* = 14.12, *p* < 0.001) in the total model, 516/661 (78.1%, *z* = 14.43, *p* < 0.001) in the positive model, and 371/661 (56.1%, *z* = 3.15, *p* = 0.002) in the negative model. In the iterations in which the difference was greater than 2 SD of life satisfaction change scores, the mean accuracy was 218/251 (86.9%, *z* = 11.68, *p* < 0.001) in the total model, 219/251 (87.3%, *z* = 11.80, *p* < 0.001) in the positive model, and 158/251 (62.9%, *z* = 4.10, *p* < 0.001) in the negative model.

The results indicated that SDNV predicted life satisfaction change scores with significant accuracy.

**Robustness across window lengths**

To test the robustness of the prediction effects across window lengths, we estimated neural variability using different window lengths (window length = 30 s, 40 s, 50 s). A previous study suggested that the results with the window lengths of approximately 30-60 s in duration were robust (Hutchison et al., 2013).

At the window length of 30 s, SDNV remained predictive of life satisfaction change scores in the total model (*r* = 0.27, *p* = 0.043, Figure S2-4) and in the positive model (*r* = 0.27, *p* = 0.042) while the prediction effect was still not significant in the negative model (*r* = -0.06, *p* = 0.650). At the window length of 50 s, SDNV remained predictive of life satisfaction change scores in the total model (*r* = 0.29, *p* = 0.029) and in the positive model (*r* = 0.29, *p* = 0.025). In the negative model, no feature was selected in at least one iteration, so the prediction analysis was not performed.

The results showed that the prediction effects were robust across window lengths.

**Robustness across feature selection thresholds**

To test the robustness of the prediction effects across feature selection thresholds, we used different thresholds ($\propto=0.10, 0.05, 0.01$) in the feature selection stage.

In the whole-brain prediction, at the feature selection threshold of $\propto=0.10$, SDNV remained predictive of life satisfaction change scores in the total model (*r* = 0.43, *p* < 0.001, Figure S2-4) and in the positive model (*r* = 0.42, *p* = 0.001) while the prediction effect was still not significant in the negative model (*r* = 0.10, *p* = 0.437). At the feature selection threshold of $\propto=0.01$, SDNV remained predictive of life satisfaction change scores in the total model (*r* = 0.39, *p* = 0.003) and in the positive model (*r* = 0.38, *p* = 0.003). In the negative model, no feature was selected in at least one iteration, so the prediction analysis was not performed.

In the network-based prediction, at the feature selection threshold of $\propto=0.10$, SDNV remained predictive of life satisfaction change scores in the default mode network (*r* = 0.49, *p* < 0.001), frontoparietal task control network (*r* = 0.48, *p* < 0.001), visual network (*r* = 0.33, *p* = 0.013), salience network (*r* = 0.33, *p* = 0.012) and memory retrieval network (*r* = 0.30, *p* = 0.023) in the positive model. The predictive effects of the remaining networks did not survive the false discovery rate (FDR) correction (Table S1). At the feature selection threshold of $\propto=0.01$, only the prediction effect of the default mode network in the positive model survived the FDR correction (*r* = 0.49, *p* < 0.001). Except for the total model at the feature selection threshold of $\propto=0.10$ in the salience network (*r* = 0.28, *p* = 0.036), in the total model or in the negative model, no feature was selected in at least one iteration, so the prediction analysis was not performed.

The results showed that the prediction effects were robust across feature selection thresholds.

**Simple effects**

In the whole-brain prediction, neural variability (node 124) was positively correlated with life satisfaction change scores (*b* = 1.15, *SE* = 0.21, *p* < 0.001, *95% CI =* [0.73, 1.56], Figure S5) in interdependent individuals (mean = 1.05) but negatively correlated with life satisfaction change scores (*b* = -0.73, *SE* = 0.20, *p* < 0.001, *95% CI =* [-1.12, -0.34]) in independent individuals (mean = -0.98) .

In the network-based prediction, neural variability within the default mode network (node 124), frontoparietal task control network (node 176), visual network (node 145) and salience network (node 218) was positively correlated with life satisfaction change scores in interdependent individuals (default mode network: *b* = 1.15, *SE* = 0.21, *p* < 0.001, *95% CI =* [0.73, 1.56]; frontoparietal task control network: *b* = 0.60, *SE* = 0.26, *p* = 0.023, *95% CI =* [0.08, 1.11]; visual network: *b* = 0.57, *SE* = 0.24, *p* = 0.024, *95% CI =* [0.08, 1.05]; salience network: *b* = 0.09, *SE* = 0.21, *p* = 0.675, *95% CI =* [-0.33, 0.51], Figure S6) but negatively correlated with life satisfaction change scores in independent individuals (default mode network: *b* = -0.73, *SE* = 0.20, *p* < 0.001, *95% CI =* [-1.12, -0.34]; frontoparietal task control network: *b* = -0.96, *SE* = 0.20, *p* < 0.001, *95% CI =* [-1.37, -0.55]; visual network: *b* = -0.40, *SE* = 0.17, *p* = 0.022, *95% CI =* [-0.73, -0.06]; salience network: *b* = -0.80, *SE* = 0.18, *p* < 0.001, *95% CI =* [-1.16, -0.44]).

**REFERENCES**

Diener, E. D., Emmons, R. A., Larsen, R. J., & Griffin, S. (1985). The satisfaction with life scale. *Journal of Personality Assessment*, *49*(1), 71-75.

Hutchison, R. M., Womelsdorf, T., Allen, E. A., Bandettini, P. A., Calhoun, V. D., Corbetta, M., Penna, S. D., Duyn, J. H., Glover, G. H., Gonzalezcastillo, J., Handwerker, D. A., Keilholz, S. D., Kiviniemi, V., Leopold, D. A., De Pasquale, F., Sporns, O., Walter, M., & Chang C. (2013). Dynamic functional connectivity: promise, issues, and interpretations. *NeuroImage*, *80*, 360-378.

Luo, S., Ma, Y., Liu, Y., Li, B., Wang, C., Shi, Z., Li, X., Zhang, W., Rao, Y., & Han, S. (2015). Interaction between oxytocin receptor polymorphism and interdependent culture values on human empathy. *Social Cognitive and Affective Neuroscience*, *10*(9), 1273-1281.

Phinney, J. S. (1992). The multigroup ethnic identity measure: A new scale for use with diverse groups. *Journal of Adolescent Research*, *7*(2), 156-176.

Power, J. D., Cohen, A. L., Nelson, S. M., Wig, G. S., Barnes, K. A., Church, J. A., Vogel, A. C., Laumann, T. O., Miezin, F. M., Schlaggar, B. L., & Petersen, S. E. (2011). Functional network organization of the human brain. *Neuron*, *72*(4), 665-678.

Singelis, T. M. (1994). The measurement of independent and interdependent self-construals. *Personality and Social Psychology Bulletin*, *20*(5), 580-591.

Yan, C., & Zang, Y. (2010). DPARSF: a MATLAB toolbox for" pipeline" data analysis of resting-state fMRI. *Frontiers in Systems Neuroscience*, *4*, 13.

Zhang, J., Cheng, W., Liu, Z., Zhang, K., Lei, X., Yao, Y., Becker, B., Liu, Y., Kendrick, K. M., Lu, G., & Feng, J. (2016). Neural, electrophysiological and anatomical basis of brain-network variability and its characteristic changes in mental disorders. *Brain*, *139*(8), 2307-2321.


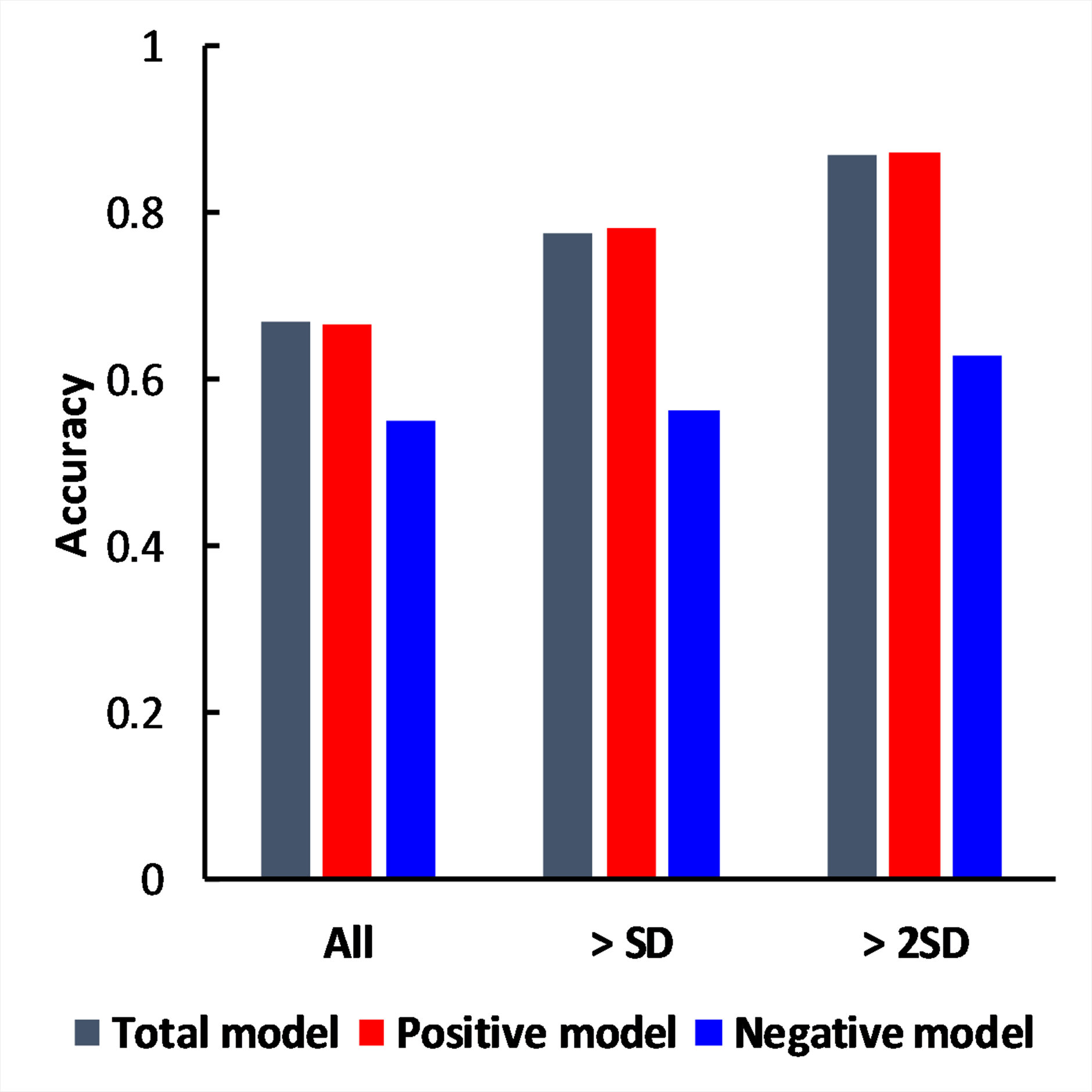


**Figure S1. Results of the leave-two-out cross-validation.** The predictive power (mean accuracy) of the total model (dark gray), positive model (red), negative model (blue) in the iterations in which the difference of life satisfaction change scores between the two participants in the test set was greater than 0 SD (all iterations), 1 SD, 2 SD of life satisfaction change scores.


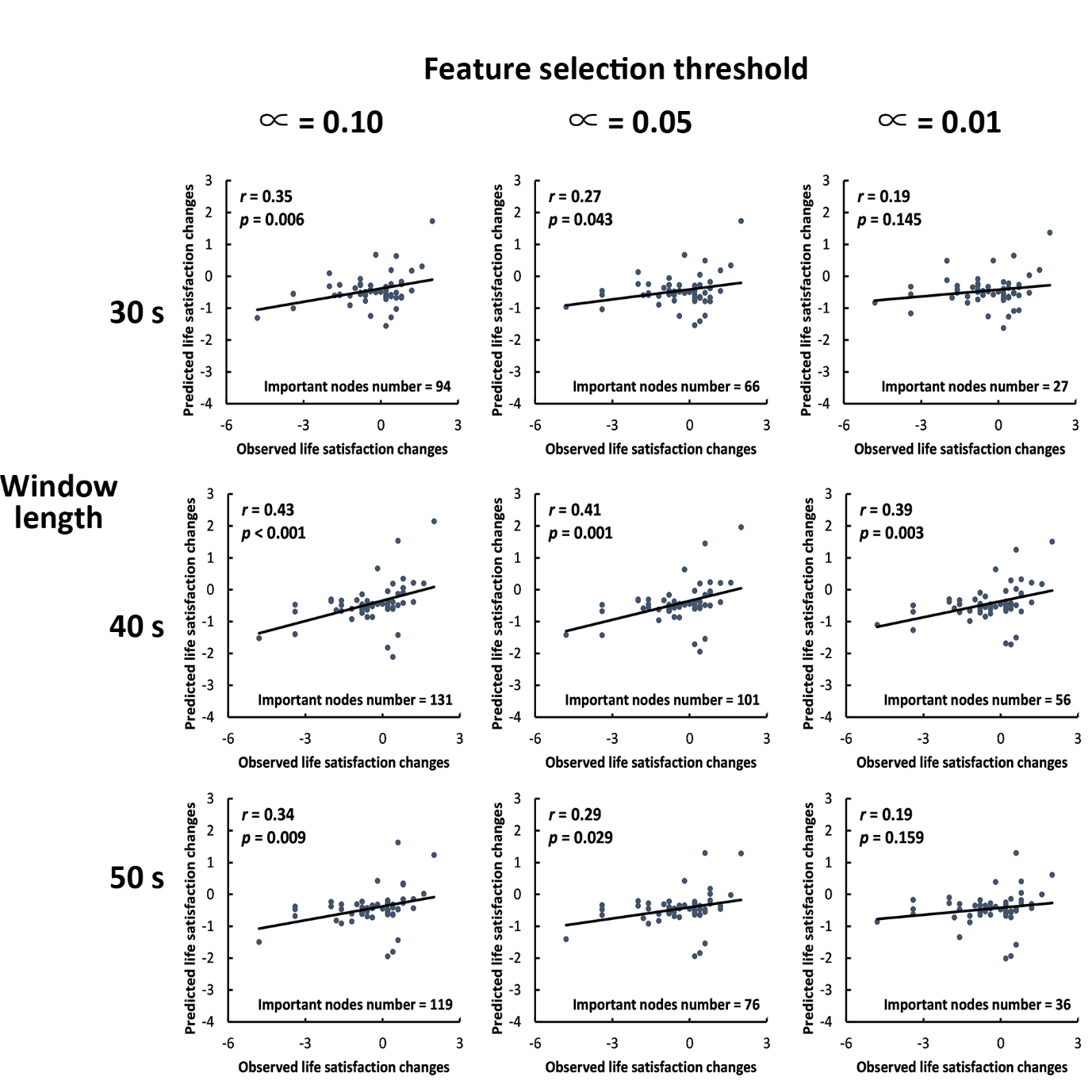


**Figure S2.** The predictive power of the total model in the whole-brain prediction at the window lengths of 30 s (up), 40 s (middle), 50 s (bottom) and at the feature selection threshold of $\propto=0.10$ (left), $\propto=0.05$ (middle), $\propto=0.01$ (right).


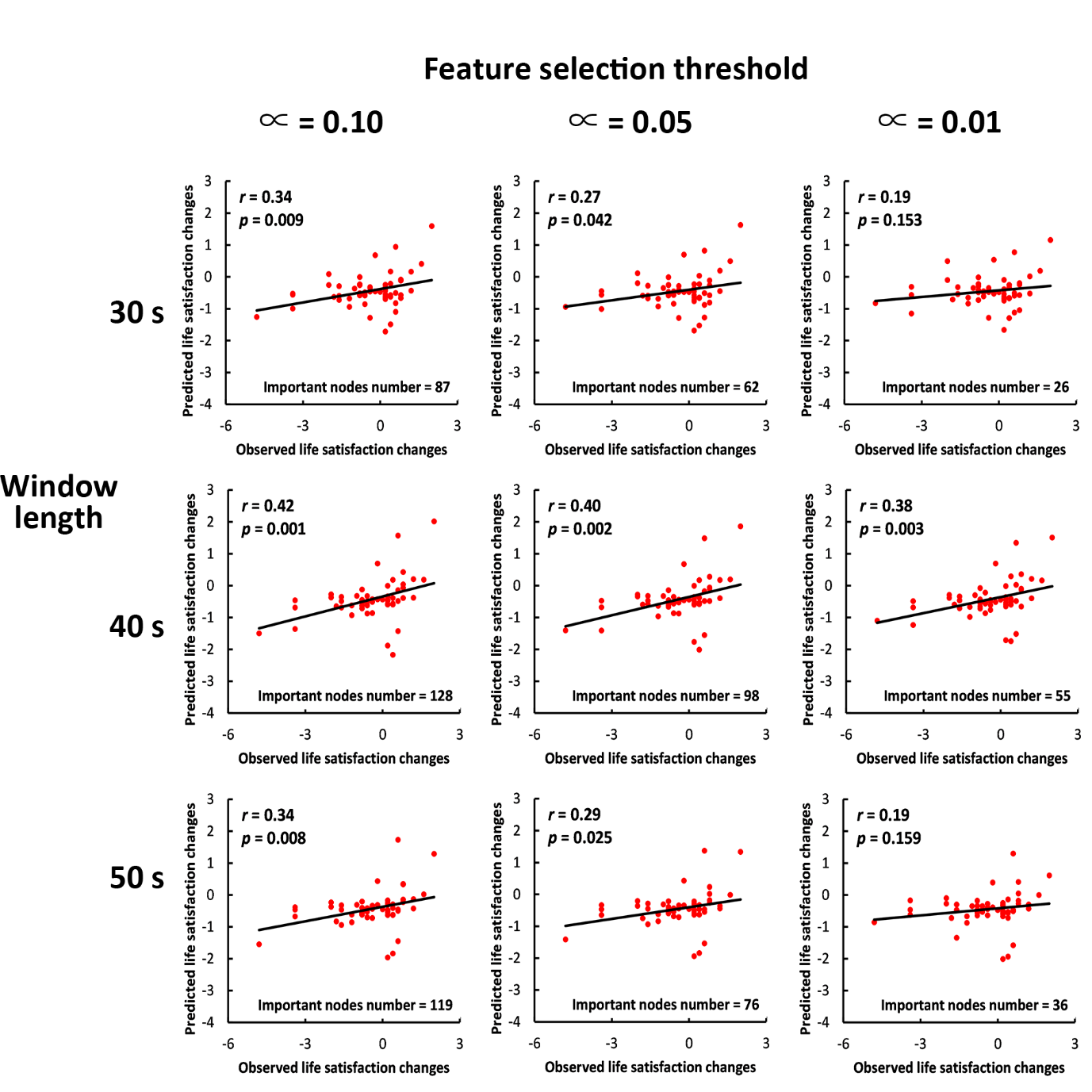


**Figure S3.** The predictive power of the positive model in the whole-brain prediction at the window lengths of 30 s (up), 40 s (middle), 50 s (bottom) and at the feature selection threshold of $\propto=0.10$ (left), $\propto=0.05$ (middle), $\propto=0.01$ (right).


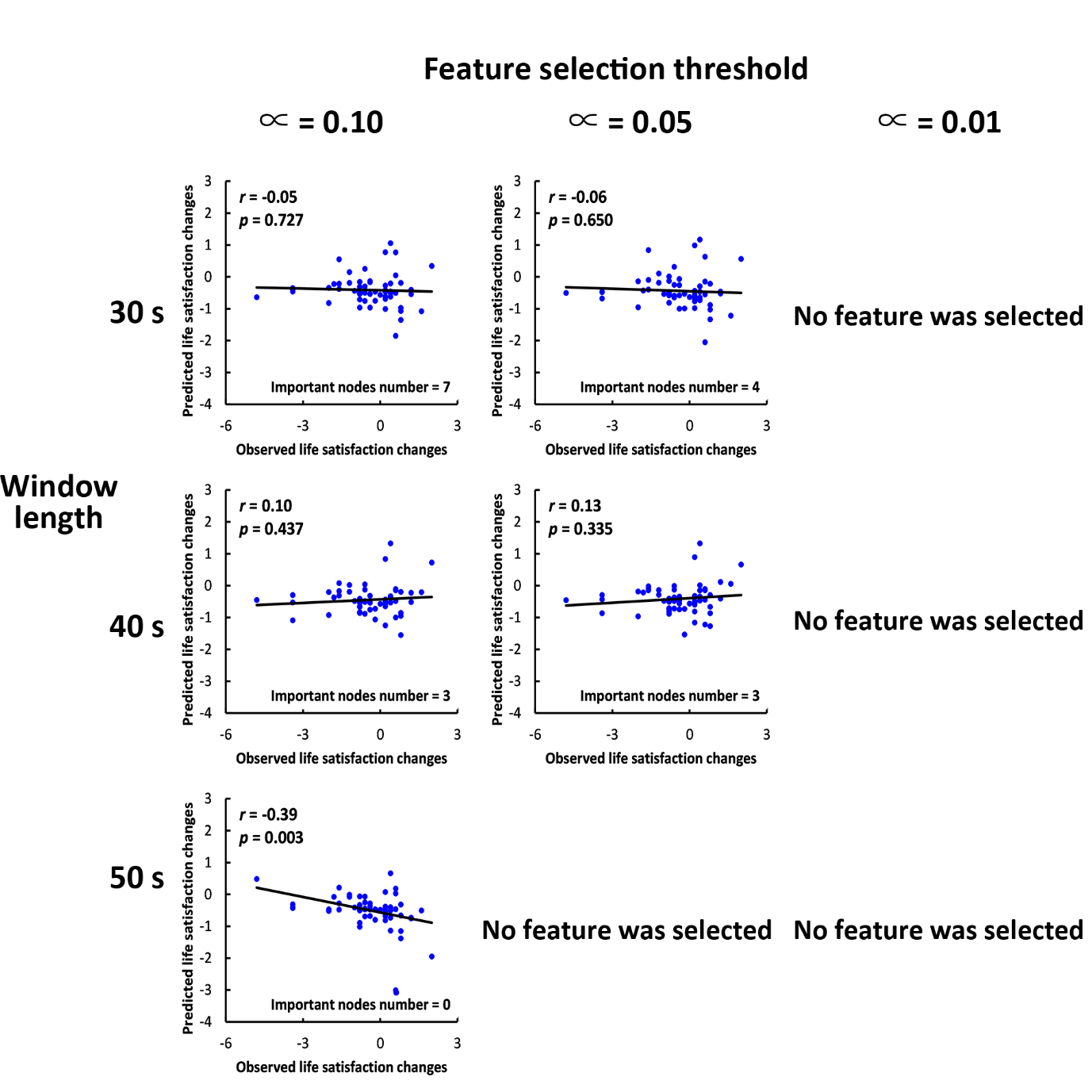


**Figure S4.** The predictive power of the negative model in the whole-brain prediction at the window lengths of 30 s (up), 40 s (middle), 50 s (bottom) and at the feature selection threshold of $\propto=0.10$ (left), $\propto=0.05$ (middle), $\propto=0.01$ (right).


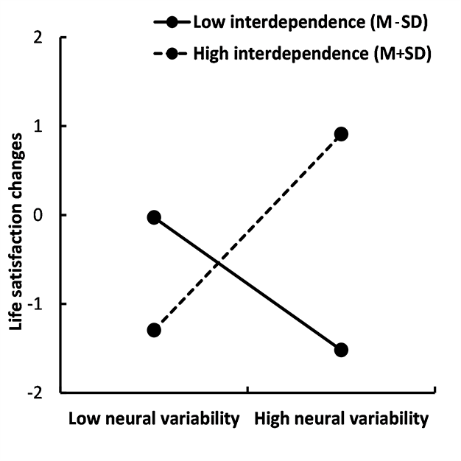


**Figure S5.** The simple effects of the whole-brain prediction using the node whose SDNV was most correlated with life satisfaction change scores.


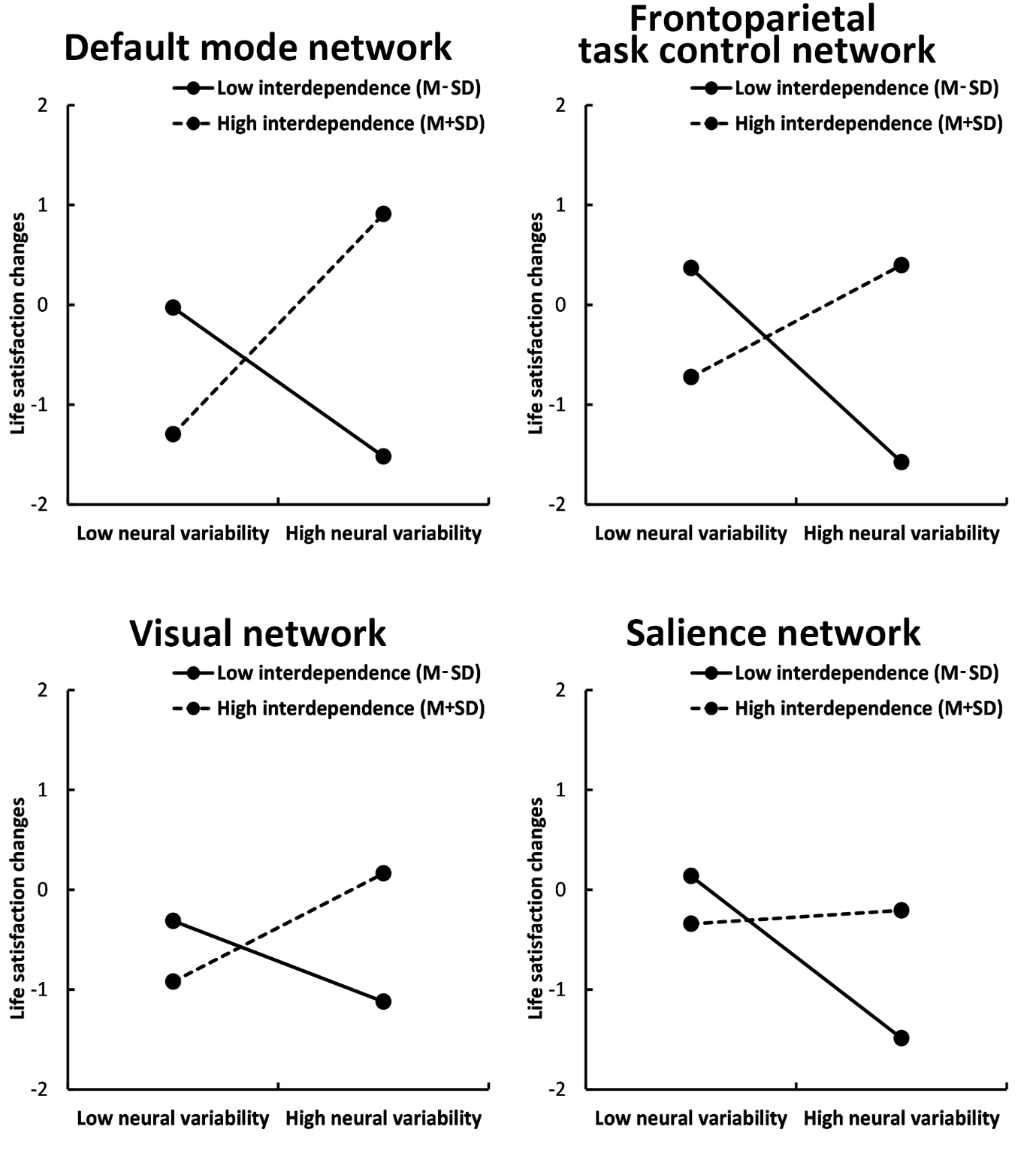


**Figure S6.** The simple effects of the network-based prediction using the node whose SDNV was most correlated with life satisfaction change scores.

**Table S1.** Network-based prediction using different feature selection thresholds.

| Networks | $\propto=0.10$ | |  |  | $\propto=0.05$ | |  |  | $\propto=0.01$ | |  |
| --- | --- | --- | --- | --- | --- | --- | --- | --- | --- | --- | --- |
|  | *r* | *p* | *INN* |  | *r* | *p* | *INN* |  | *r* | *p* | *INN* |
| Default mode network | 0.49 | <0.001* | 41 |  | 0.48 | <0.001* | 36 |  | 0.49 | <0.001* | 20 |
| Frontoparietal task control network | 0.48 | <0.001* | 13 |  | 0.38 | 0.004* | 10 |  | 0.30 | 0.022 | 6 |
| Visual network | 0.33 | 0.013* | 22 |  | 0.32 | 0.014* | 16 |  | 0.21 | 0.115 | 10 |
| Salience network | 0.33 | 0.012* | 4 |  | 0.31 | 0.018* | 4 |  | 0.18 | 0.184 | 2 |
| Dorsal attention network | 0.25 | 0.056 | 4 |  | 0.27 | 0.043 | 3 |  | - | - | 2 |
| Memory retrieval network | 0.30 | 0.023* | 4 |  | 0.26 | 0.045 | 3 |  | 0.22 | 0.093 | 1 |
| Cingulo-opercular task control network | 0.19 | 0.149 | 8 |  | 0.19 | 0.163 | 5 |  | 0.10 | 0.457 | 2 |
| Hand sensory-somatomotor network | 0.18 | 0.177 | 6 |  | 0.14 | 0.299 | 5 |  | -0.06 | 0.660 | 2 |
| Subcortical network | 0.11 | 0.401 | 5 |  | 0.04 | 0.756 | 3 |  | - | - | 0 |
| Ventral attention network | 0.16 | 0.219 | 5 |  | -0.07 | 0.608 | 1 |  | - | - | 0 |
| Auditory network | - | - | 0 |  | - | - | 0 |  | - | - | 0 |
| Mouth sensory-somatomotor network | - | - | 0 |  | - | - | 0 |  | - | - | 0 |
| Cerebellar network | - | - | 0 |  | - | - | 0 |  | - | - | 0 |

Note. *INN* referred to important nodes number. * fdr corrected *p* < .05.

**Table S2.** Correlation between head motion and life satisfaction change scores.

| Head motion parameters | Pearson correlation coefficient | Significance |
| --- | --- | --- |
| Parameter 1 (translation) | -0.13 | 0.348 |
| Parameter 2 (translation) | 0.17 | 0.196 |
| Parameter 3 (translation) | -0.10 | 0.475 |
| Parameter 4 (rotation) | 0.03 | 0.798 |
| Parameter 5 (rotation) | -0.17 | 0.193 |
| Parameter 6 (rotation) | 0.06 | 0.664 |
| Maximum of all parameters  (parameter 1-6) | 0.02 | 0.856 |
| Maximum of translation parameters  (parameter 1-3) | -0.05 | 0.739 |
| Maximum of rotation parameters  (parameter 1-3) | 0.06 | 0.677 |
| Sum of all parameters  (parameter 1-6) | 0.00 | 0.997 |
| Sum of translation parameters  (parameter 1-3) | 0.00 | 0.986 |
| Sum of rotation parameters  (parameter 1-3) | 0.00 | 0.983 |
|  |  |  |
| Framewise displacement | -0.01 | 0.942 |
